# The Role of MDCT Coronary Angiography in the Detection of Benign Varieties and Anomalies of Coronary Blood Vessels—A Narrative Review

**DOI:** 10.3390/medicina61040765

**Published:** 2025-04-21

**Authors:** Ana Mladenovic Markovic, Ana Tomic, Miodrag Nisevic, Biljana Parapid, Nikola Boskovic, Marina Vitas, Miona Jevtovic, Sandra Grujicic

**Affiliations:** 1 Centre for Radiology and Magnetic Resonance Imaging, University Clinical Centre of Serbia, Pasterova 2, 11000 Belgrade, Serbia; amladenovicmarkovic@gmail.com (A.M.M.); anatomic9977@gmail.com (A.T.); miodrag.nisevic@gmail.com (M.N.); mionajevtovic1@gmail.com (M.J.); 2 Faculty of Medicine, University of Belgrade, Dr Subotica Starijeg 8, 11000 Belgrade, Serbia; biljana_parapid@yahoo.com; 3 Clinic for Cardiology, University Clinical Centre of Serbia, Pasterova 2, 11000 Belgrade, Serbia; belkan87@gmail.com; 4 Institute of Mother and Child Health Care of Serbia “Dr Vukan Čupić”, 11000 Belgrade, Serbia; mvitas246@gmail.com; 5 Institute of Epidemiology, Faculty of Medicine, University of Belgrade, Dr Subotica Starijeg 8, 11000 Belgrade, Serbia

**Keywords:** coronary vessels, coronary variations, coronary anomalies, MDCT coronary angiography, diagnosis

## Abstract

Coronary arteries may vary in quantity, point of origin, or course. These variations fall under the category of anatomical variants/anomalies of the coronary arteries, representing congenital abnormalities of the coronary vascular system. Generally, they are benign, asymptomatic, and identified inadvertently during coronary angiography conducted for alternative indications. However, in some cases, the anomaly’s characteristics or its interaction with surrounding structures may cause hemodynamic disturbances. These disturbances can lead to turbulent blood flow, which in turn poses an increased likelihood for the development of atherosclerosis and myocardial ischemia. If symptomatic, potential manifestations include chest pain, arrhythmias, syncope, myocardial infarction, and sudden cardiac death. Given the potential for life-threatening complications in certain cases, the early and accurate diagnosis of coronary artery anomalies is of paramount importance. The most common diagnostic procedures used for the evaluation of coronary vessels are coronary angiography and multi-detector row computed tomography (MDCT) coronary angiography. MDCT angiography is a non-invasive, dependable, safe, and sensitive method for the detailed visualization of coronary anatomy. It offers high-resolution imaging that enables precise assessment of congenital coronary variations, aiding in both clinical decision-making and long-term patient management. We conducted a narrative review to analyze and integrate the body of literature on coronary artery varieties and anomalies. Our objective was to provide a comprehensive, albeit non-exhaustive, overview of essential concepts and findings related to their definition, classification, and detection with MDCT angiography. By integrating current knowledge in MDCT imaging, we seek to contribute to a better understanding of the clinical implications of coronary artery variations and their role in cardiovascular health.

## 1. Introduction

Coronary artery anomalies and their variants are rare congenital conditions classified into three varieties, based on their origin, number, and path. Depending on the population under consideration, the prevalence of this condition ranges from 0.3% to 2.6% [1,2,3]. The prevalence of coronary artery variations is predominantly attributed to variations in their origin and distribution, constituting over 95% of all anomalies affecting the coronary vessels [4]. They are defined based on the anatomical characteristics of any of the three main epicardial coronary arteries (CA): the left anterior descending artery (LAD), left circumflex artery (LCX), and right coronary artery (RCA). An anomaly is detected by divergence from typical morphological characteristics [5,6]. In defining an abnormal CA, one must include all observed variations in the anatomic features that describe the coronary arteries (Table 1). Anomalies can affect the artery from the ostium (or the point of origin) along its subsequent course and branching.

### Symptoms and Clinical Significance of Coronary Vessel Anomalies

Most anomalies and varieties of CA are asymptomatic incidental findings on routine coronary angiography as a diagnostic workup for ischemic heart disease [7,8]. Hemodynamic disturbances may occur due to the interaction of the anomaly with its surrounding structures (such as the myocardium or pericardium) and its associated characteristics. These disturbances can lead to turbulent blood flow, increasing the likelihood of atherosclerosis development [9].

Certain anomalies in the position and orientation can cause significant circulatory disorders. Their clinical significance is evident in the increased risk of myocardial ischemia, typically during physical exertion, due to diminished blood flow. Consequently, individuals may experience symptoms such as chest pain and syncope. Rarely, this can lead to myocardial infarction, heart failure, or sudden cardiac death (SCD), particularly in young adults [10,11]. Anomalies in the coronary blood vessels pose a significant challenge for cardiologists during catheterization, which is evident when manipulating catheters and wires in areas where anomalies are present [12].

Noninvasive imaging techniques, such as computed tomography coronary angiography (CTCA), have facilitated the precise visualization of coronary artery anomalies related to their origin, course, and termination [13]. Multi-detector row computed tomography (MDCT) is considered more effective than conventional angiography for accurately identifying an anomalous coronary artery’s starting point and initial course [13,14]. Consequently, MDCT is considered the optimal method for conducting a comprehensive three-dimensional assessment of the coronary tree.

This review establishes the significance of MDCT coronary angiography in identifying anatomical variations and abnormalities in coronary blood vessels while also highlighting its advantages over conventional coronary angiography. Additionally, it provides clinical perspectives emphasizing the significance of documenting these disorders in radiology reports.

## 2. Literature Search

This review follows a narrative, unsystematic approach to examine and synthesize the existing literature on the aforementioned topic, providing a comprehensive, but non-exhaustive, overview of key concepts, trends, and findings from relevant sources. To identify all the relevant papers about detection of different coronary artery anomalies on MDCT, a PubMed literature search was performed. Keywords and phrases related to “MDCT”, “coronary anomalies”, and “coronary variety” were used to identify relevant studies. The search strategy for this narrative review was pre-planned, employing comprehensive methods to identify all relevant studies. Any iterative adjustments made during the review process were driven by emerging themes and constructs, rather than by a search for new concepts alone. The last search was performed on 2 September 2024. Filters were applied to consider only articles published in English. The inclusion criteria included studies that used MDCT to detect and/or present cases of different CA anomalies and varieties. The exclusion criteria included articles that were not written in English, as well as articles that contained technical assessments, along with a lack of established diagnostic methods and a failure to adequately report outcomes related to anatomical variations. This review was structured around established frameworks for the use of MDCT in coronary angiography, with the analysis aimed at either affirming or challenging these predefined themes, with the exception for the coiling of the coronary arteries, as a variant of the pathway not yet clearly defined in the available literature. We approached the literature with specific constructs in mind, assessing the studies to determine how they aligned with these established categories. MDCT images of coronary artery anomalies and variations were retrospectively gathered from our department’s library of MDCT coronary angiography procedures for the presentation of relevant cases of coronary anomalies. As this study was conducted as a narrative review, the process of identifying emerging themes followed an iterative and interpretative approach. Initially, the relevant literature was reviewed based on its conceptual alignment with the objectives of the review. The selection of the literature was guided by its relevance to the clinical understanding, diagnosis, and management of coronary artery anomalies. During the review process, key themes began to emerge through repeated reading and the comparison of findings across multiple sources. Importantly, the thematic framework was also shaped by our clinical experience and exposure to cases managed within our department. These real-world cases highlighted specific diagnostic challenges, anatomical variants, and management considerations that informed the focus areas of the review.

Therefore, in this review, we establish the significance of MDCT coronary angiography in identifying anatomical variations and abnormalities in coronary blood vessels, while also highlighting its advantages over conventional coronary angiography. Additionally, we provide clinical perspectives that emphasize the significance of documenting these disorders in radiology reports.

## 3. Varieties of Coronary Arteries

Including an anomalous origin of coronary anatomy in the interpretation is imperative because of its significant implications for patients, cardiologists, and surgeons [8]. We divide them into varieties based on the origin, number, and pathway.

### 3.1. Origin Varieties of Coronary Arteries

Various factors, including the number, position, dimensions, and angle of origin, can determine the classification of abnormalities in the ostia of arteries [15].

#### 3.1.1. The Absence of Left Main Artery LMA

**Figure 1:**
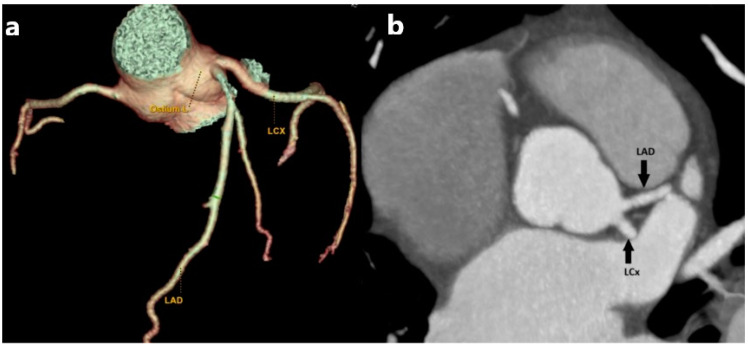
MDCT images presenting an absent LMA. (**a**) Volume-rendered (VR) MDCT images show an absent LMA with separate origins of LCX and LAD from the left sinus of Valsalva. (**b**) Planar view of LAD and LCX with its own separate origin from the left sinus (black arrow). This variant, although often asymptomatic, is important to recognize preoperatively or prior to coronary interventions to avoid complications during catheterization or surgery.

The LMA originates in the left sinus of Valsalva. It soon branches into the LAD and LCX, which supply the left ventricular wall and most of the interventricular septum (Figure 1). This specific coronary variation occurs when the LMA is congenitally absent. Consequently, the LAD and LCX originate separately from the left sinus of Valsalva [16]. It is rare and remains asymptomatic in most cases [17]. Some patients present with chest pain upon exertion, palpitations, and syncope. Angina and arrhythmia manifestations should be assessed cautiously, and the vigilant monitoring of symptoms is necessary to respond to escalation promptly. During catheterization, angiography of the CA, LAD, and LCX arising separately from the left sinus of Valsalva can be easily misdiagnosed as a coronary occlusion or atresia if the operator evaluates only one orifice at a time. MDCT can accurately provide crucial diagnostic information about the origin, course, and anatomical relationships of the coronary arteries and thus accurately detect congenital anomalies of the coronary vessels [18].

#### 3.1.2. Aberrant Origin of the RCA, LMA, or Its Branches

**Figure 2:**
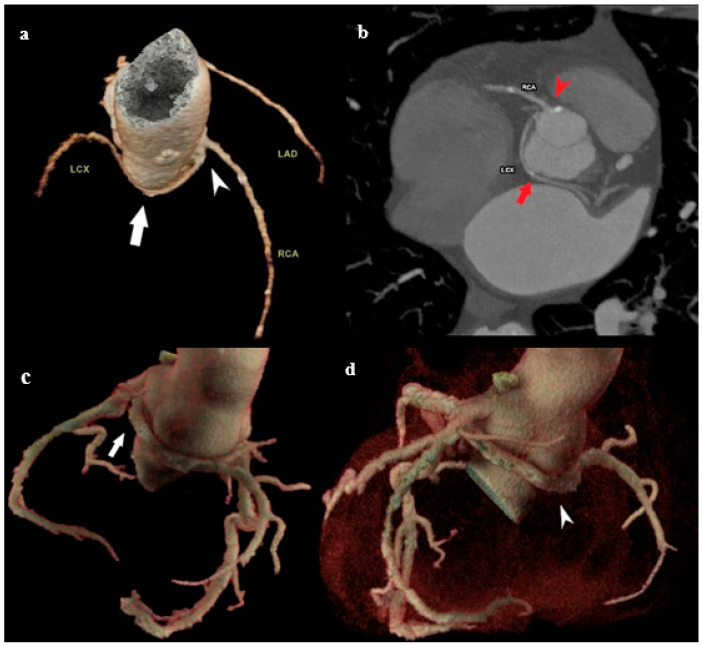
Patients with aberrant origin of the coronary arteries. (**a**) Volume-rendered (VR) MDCT image of posterior view of the LCX arising from the right sinus of Valsalva (white arrow head), with its retrobulbar course (white arrow). (**b**) MDCT planar (axial) view of the LCX arising from the right sinus of Valsalva (red arrow head), with its retrobulbar course, moving between the aortic root and left atrium (red arrow). (**c**,**d**) MDCT image of posterior view of the single ostia and RCA arising from the left sinus of Valsalva (arrow in (**c**)), with retrobulbar position and further regular course (arrow head in (**d**)). With the noted retrobulbar course, these anomalies may occasionally lead to ischemia, especially if the vessel is compressed between great vessels, warranting further evaluation.

The anomalous origin of the coronary arteries is mostly an asymptomatic anomaly, with the exception of some rare cases when angina, syncope, heart failure, and sudden death may occur. The anomalous origin of LCX from the right coronary sinus is a prevalent congenital coronary variation (Figure 2a,b), with a documented prevalence of 0.18–0.67% in the literature [19]. It is typically a benign medical condition, but, in rare instances, individuals may experience atypical chest pain and discomfort [20].

Conversely, an aberrant origin of the LMA is a rare anomaly with a much more serious prognosis, often accompanied by angina complaints, myocardial infarction, and sudden death in asymptomatic patients.

White and Edwards first described an aberrant origin of the RCA in 1948 [21]. The anomalous origin of the RCA from the left sinus of Valsalva (Figure 2c,d) is a rare condition reported in 0.43% of patients undergoing MDCT coronary angiography [22], occurring more often in Hispanic individuals (0.25% of cases). This condition is usually asymptomatic but can be accompanied by anginal complaints, usually during exertion or rhythm disturbances [23].

The normal origins of the RCA and LMA are the right and left sinuses of Valsalva, respectively. Atypical origins, in which a blood vessel on the right or left side of the heart is separated from the wrong coronary sinus, can be classified into several subcategories, as presented in Table 2.

Developed symptomatology and myocardial ischemia are first caused by anomalies that have a course between the pulmonary artery and the aortic bulb, primarily due to their path between two large blood vessels that exert pressure on the coronary blood vessel, the angle that the blood vessel makes on its way, and, either immediately after the origin passes, or due to the passage of the coronary artery itself through the aortic wall, the existence of an intramural segment, which can lead to its spasm. The best way to show this variety is MDCT coronary angiography, which shows not only the abnormal origin but also the relationship to other structures (large blood vessels) and the existence of an intramural segment.

#### 3.1.3. Acute Takeoff of the Coronary Arteries

**Figure 3:**
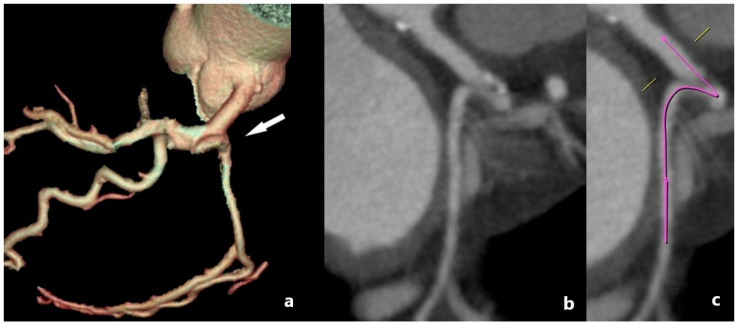
MDCT angiography of acute takeoff anomaly. (**a**) VR MDCT image of acute takeoff of the LCX (arrow). (**b**,**c**) Planar view of the acute origin of the LCX, with a denoted artery pathway marking the separation of the LCX at an angle of less than 45°. This kind of an acute takeoff angle can increase susceptibility to atherosclerosis and may be associated with myocardial ischemia or compromised perfusion under stress.

Acute takeoff is the separation of a blood vessel at an angle of less than or equal to 45 degrees. It was first described by Angelini et al. [24] and refers to the separation of the LCX (Figure 3). This variation is observed in approximately 2% of the population and can be a problem in interventional radiological procedures, with an increased chance of unsuccessful intervention.

In a postmortem study in 1984, Virmani et al. performed an autopsy to investigate the cause of death in 22 patients in whom the cause of death was unknown and compared it with 19 patients who died of known causes. The hearts of these 41 patients were examined for abnormalities in the acute angle take-off of the coronary artery and the presence of ostial valve-like ridges. Patients who died suddenly had acute angle takeoff of the coronary artery. The study proposed that the coronary artery forms an acute angle at its origin with the aortic wall as the aortic root dilates the ostial valve-like ridge, compressing the ridge against the coronary artery wall. In addition, it is possible that diminished flow occurs in the abnormally angled coronary artery and that the ostial valve-like ridge impedes flow into the coronary artery by obstructing the ostium [25].

#### 3.1.4. Coronary Arteries High Takeoff

**Figure 4:**
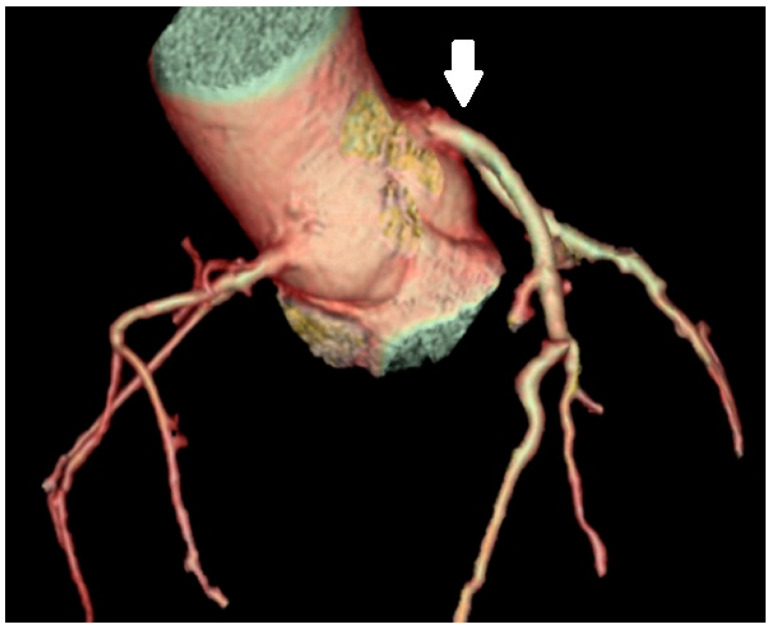
VR MDCT image showing a high angle takeoff (above the sinotubular junction) of the RCA (arrow). In this case, the high takeoff of the RCA may complicate catheter engagement during angiography and increase the risk of ischemia due to lengthened course.

High take-off of the coronary artery is a rare anomaly that arises more than 10 mm above the sinotubular junction [26]. It is a rare anatomical variation that can occur alone or with other congenital heart defects [27]. The reported incidence of RCA high takeoff (Figure 4) is 0.02% to 0.2% [3].

It is often asymptomatic and is found only during surgery or autopsy. The main problem is reduced coronary perfusion, which leads to myocardial ischemia, angina, syncope, or sudden death, even in the absence of atherosclerosis. This anomaly is usually asymptomatic in children and is an incidental finding during diagnostic tests, cardiac surgery, or correction of other heart defects. High RCA take-off also impacts surgical procedures. When performing procedures on a patient with this anomaly, anatomical variations increase the technical challenges, such as the unsuccessful induction of cardiac arrest. For the precise diagnosis of this variation, MDCT is suggested, especially in adult patients. In symptomatic adult patients, the anomaly can be corrected surgically using a saphenous vein graft or by reimplanting the RCA into the correct sinus [28].

#### 3.1.5. A Single Coronary Artery/Ostia

**Figure 5:**
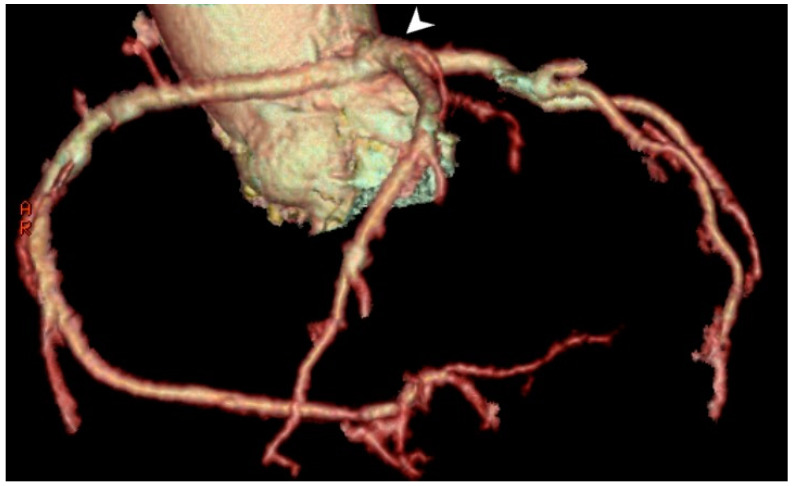
MDCT coronary angiography revealed a single coronary artery, originating from an ostium in the left sinus of Valsalva (white arrow head). This rare anomaly may range from benign to life-threatening depending on the artery’s course—especially if it passes between the aorta and pulmonary artery.

A single coronary artery is a congenital variety that can be classified according to its origin and number. A single coronary artery/ostia is a rare anomaly in which only one blood vessel separates from the left or right coronary sinus and splits into branches for the right and left halves of the heart (Figure 5). Patients with single CA are typically asymptomatic. In some cases, the path of one of the vessels is between the aortic bulb and the pulmonary trunk when anginal complaints occur due to pressure on the blood vessel. Other anomalies, such as the intramural course, acute take-off angle of the anomalous vessel, and a slit-like orifice, can predispose patients to anginal symptoms and sudden death during exertion.

A solitary coronary artery/ostia was first described by Hyrtl in 1841 [29], who described a single coronary artery in the absence of a left or right coronary artery; that is, the existence of a single blood vessel that supplies blood to the entire heart. Smith (1950) first classified these into the following three groups: (1) consisting of a single left or right coronary artery; (2) patients in whom an artery was isolated from one ostium and then divided into branches, one of which would cross to the opposite side of the heart and follow the path of the missing coronary artery; (3) atypical cases that could not be classified in the first or second group [30]. Surgical options include osteoplasty, coronary artery bypass grafting (CABG) of the anomalous artery, and the re-implantation of the anomalous artery into the aorta.

### 3.2. Number Variators

#### 3.2.1. Left Main Artery (LMA) Trifurcation and Quadfurcation

**Figure 6:**
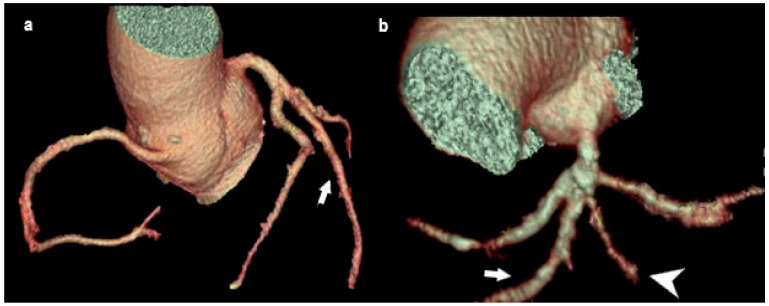
(**a**) MDCT coronary angiography showing trifurcation of LMA into LAD, LCX, and ramus intermedius (white arrow). (**b**) MDCT coronary angiography showing quadrifurcation with ramus intermedius (white arrow) and obtuse marginal branch (white arrow head). Understanding these variants is essential during revascularization procedures or coronary artery bypass grafting (CABG) to ensure all branches are adequately treated.

The left coronary artery (LCA) typically originates from the left coronary sinus and left main coronary artery (LMA). It is smaller, measuring 5 to 10 mm long, bifurcating into the left anterior descending (LAD) and circumflexing (LCX) arteries [31]. The most common variation in the LMA anatomy is its third branch, the ramus intermedius artery, also called LMA trifurcation (Figure 6a), or less commonly, the fourth, obtuse marginal branch (OM), an LMA quadfurcation (Figure 6b) [32]. The ramus intermedius has variable branching and may extend diagonally, following the course of the LAD, or as a marginal branch, following the course of the LCX, depending on whether it supplies the anterior or lateral wall [33]. The peculiarity of this vessel is that it does not move along the anatomical groove but slides along the free surface of the left ventricle [34].

Based on different studies, the prevalence of this anomaly ranges from 12% [35] to 30% [36] in the general population and is typically asymptomatic. The predilection site for the onset of atherosclerotic disease is commonly observed at a wider angle of the LMA branching (trifurcation or quadfurcation) [9]. This observation is of significant clinical importance because of the previously mentioned rather high frequency of this anomaly. LMA trifurcation stenosis is a challenging lesion subset for percutaneous coronary intervention with its intrinsic anatomical complexity (three branches, at least four angles, wide variability in branch size, and disease) [37,38,39]. Therefore, the preprocedural evaluation of MDCT is crucial when planning coronary catheterization.

#### 3.2.2. Double Right Coronary Artery (RCA)

**Figure 7:**
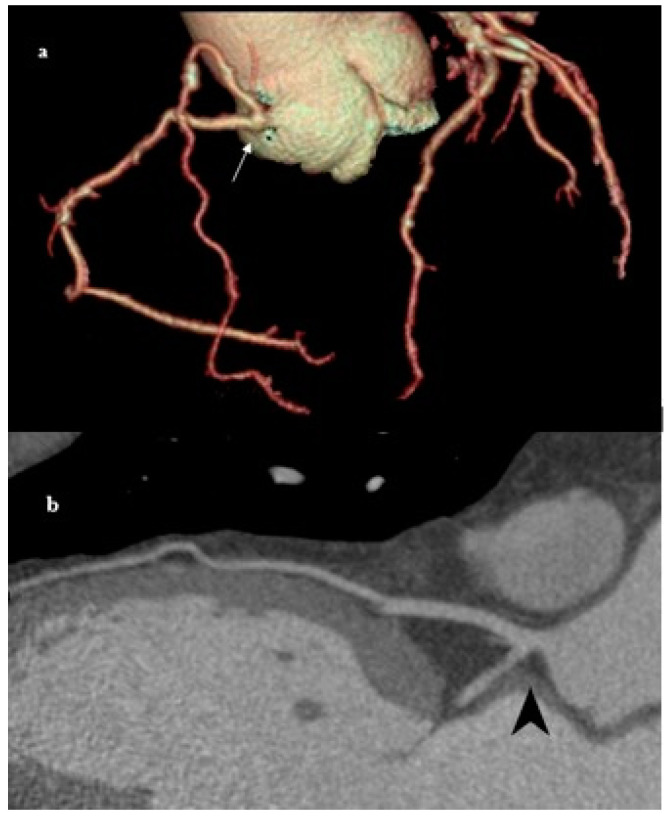
Double RCA. (**a**) MDCT coronary angiography revealed two separate RCAs originating from a single ostium in the right sinus of Valsalva (arrow). Both RCAs gave off branches with typical courses and in parallel distribution. (**b**) Planar view of two separate RCAs originating from a single ostium in the right sinus of Valsalva (black arrow head). Though usually benign, this variation must be clearly identified to avoid misinterpretation during angiography and to guide stent placement or surgery.

A double right coronary artery is defined as two right coronary arteries of similar diameters and flows arising from one or two separate openings in the right sinus of Valsalva (Figure 7), generally having complete regular branches that occur in single RCAs, each of which has its posterior descending artery (PDA) in its distal course [40]. Double RCA is an extremely rare coronary anomaly, with a prevalence ranging from 0.07 to 0.46%, depending on the report [41,42]. This variation is primarily benign and is discovered incidentally during radiological diagnosis in patients with cardiorespiratory symptoms. However, it is a predilection site for atherosclerosis, life-threatening arrhythmias, and myocardial infarction. In addition, it sometimes presents challenges to performing interventional coronary procedures [43]. The exact diagnosis of a double RCA cannot be readily established based on conventional coronary angiography, because it is difficult to distinguish this variation from the variation in the high branching of the right coronary artery. For this purpose, MDCT coronary angiography is the method of choice to differentiate between these aberrations.

#### 3.2.3. Hypoplastic Coronary Arteries (LAD, LCX, RCA)

A hypoplastic blood vessel indicates a lumen narrowing or a short course. This phenomenon can congenitally occur in all coronary arteries. Almost all types of coronary hypoplasia initially present as syncope, angina, sudden cardiac death, or incidental findings on imaging.

The LMA rapidly bifurcates into the LAD and LCX. In most people, the LAD remains the dominant vessel and supplies approximately two-thirds of the heart. However, in rare cases, the LAD remains rudimentary, which consequently increases the risk of myocardial ischemia [44]. Hypoplasia is defined by the short course and narrow vessel lumen of all or part of the left anterior descending artery and can be demonstrated using CTCA or invasive coronary angiography [45].

A rudimentary RCA is an underdeveloped RCA with a narrow lumen or a short course. The clinical conditions associated with a hypoplastic RCA include LCA dominance, LMA absence, and sudden cardiac death. Hypoplastic RCA can be an incidental finding on invasive coronary angiography computed tomography (CT) or cardiac magnetic resonance imaging MR, and the initial manifestation is syncope or sudden cardiac death. The differential diagnosis of a rudimentary RCA includes RCA stenosis or complete occlusion [46].

**Figure 8:**
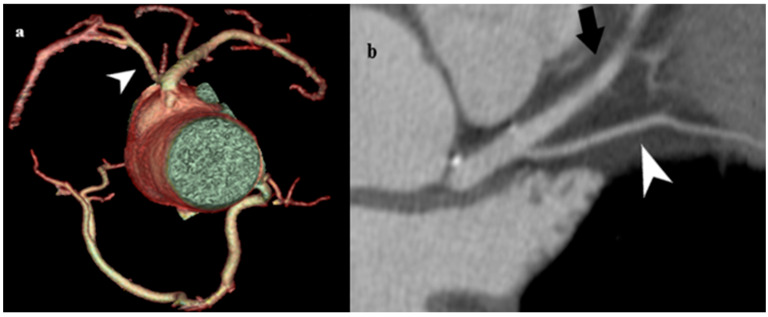
Rudimentary LCX. (**a**) MDCT coronary angiography showing rudimentary LCX (white arrow head). (**b**) Planar view of rudimentary LCX (white arrow head), with left anterior descending (LAD) artery (black arrow). A hypoplastic or rudimentary LCX often results in right-dominant circulation, as can be seen in this case. It may be associated with localized ischemia if collateral flow is inadequate.

Rudimentary LCX (Figure 8) is a rare phenomenon reported in the literature [47,48]. The most common symptom of a hypoplastic or absent LCX is chest pain on exertion, which is explained by the ‘steal phenomenon’; due to increased demand in the area normally supplied by the LCX, transient ischemia occurs in the basins supplied by the LAD and the RCA. This is a benign incidental finding. However, some patients have angina-like symptoms that often result in the detection of this rare anatomy on coronary angiography [49].

### 3.3. Aberrant Coronary Artery Pathway

#### 3.3.1. Coronary Vessel Coiling

**Figure 9:**
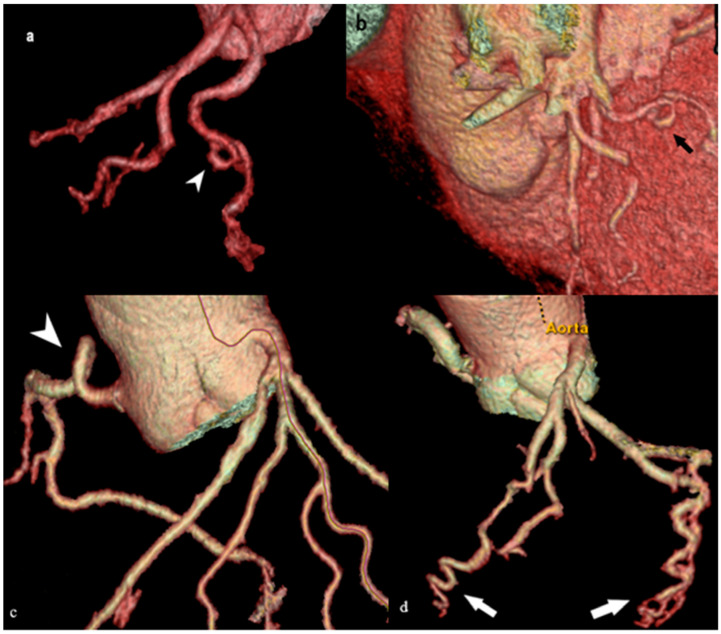
Aberrant coronary artery pathway. (**a**) MDCT VR image showing the coiling of the LCX (white arrow head). (**b**) VR MDCT image showing the LCX coiling from a different angle (black arrow), along with the accompanying cardiac anatomy. (**c**) MDCT VR image showing kinking of the RCA in proximal segment (white arrow head). (**d**) The corkscrew sign (white arrows). These tortuous courses can pose technical challenges during catheter-based procedures and may predispose patients to ischemia or complicate stent delivery.

Coronary vessel coiling (Figure 9a,b) has yet to be defined as an imaging entity in the literature. This condition is presented as a 360° angulation or bending of the artery; that is, a full circle, after which the artery continues in the original direction. It is an extremely rare finding, and, in the literature, is described as an extreme tortuosity—a 360° coronary loop around an arbitrary line passing throughout the tortuosity [50]. In our 15 years of experience, we have detected two cases. Both reported cases were identified in older adults; therefore, the blood vessel presumably elongates with tunica media thinning over time. The blood flow through a blood vessel with coiling is turbulent and can lead to plaque formation, which is also a risk factor for coronary ischemia.

#### 3.3.2. Tortuosity of Coronary Blood Vessels and Corkscrew Appearance

The existence of three or more consecutive angulations from 90° to 180° is considered a coronary artery torus, as well as a transverse diameter of two or more millimeters (Figure 9c). A more severe type of tortuosity is the existence of two or more 180° angulations in a blood vessel with a diameter greater than 2 mm. The corkscrew sign (Figure 9d), the spiral course of a coronary artery more than 360° perpendicular to the epicardial plane, is a common form of tortuosity in the coronary blood vessels. There are indications that precisely this form of coronary blood vessels may be the cause of spontaneous coronary artery dissection (SCAD). SCAD causes acute coronary syndrome in blood vessels that are not atherosclerotic and is more common in younger women [51].

#### 3.3.3. Myocardial Bridge

**Figure 10:**
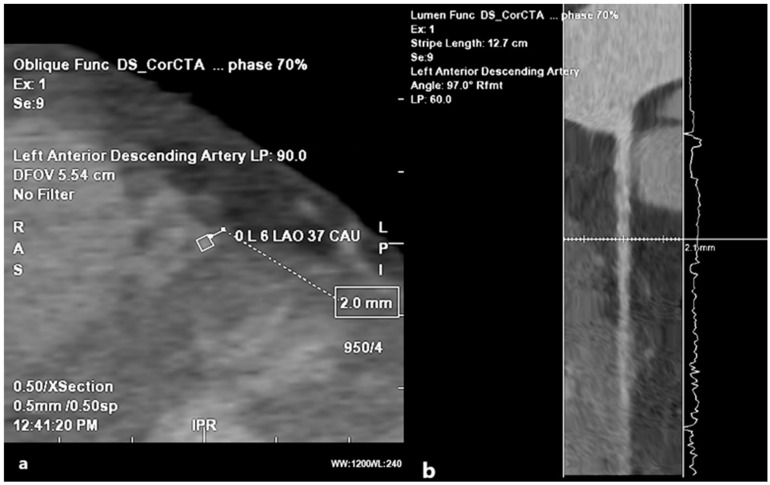
Myocardial bridging. (**a**) MDCT coronary angiography 2D planar view showing artery kinking into the myocardium (lumen labeled in rhomboid shape), at a depth of 2 mm. (**b**) Angiographic view of the myocardial bridging.

The coronary arteries have a typical epicardial path. Myocardial bridging (MB) represents the sinking of the coronary artery into the muscle, followed by resurfacing, and in most cases, is benign. Depending on the number of fibers that pass over the coronary artery, we distinguish between superficial MB (1–2 mm of the myocardium) and deep MB (>2 mm of the myocardium) bridges. This distinction is relevant because the depth is proportional to the compression exerted in systole; superficial bridges are typically asymptomatic [52]. We can talk about MB as a benign anomaly only if its depth does not exceed 2 mm (Figure 10); that is, if it is a superficial MB, not only the depth at which the coronary artery is located, but also the length of the segment that has intramural flow and the number of branches affected by the MB, will influence the onset of symptoms.

In this intramyocardial segment (segment within the heart wall), the systolic compression of a tunneled coronary artery may cause symptoms that disappear during diastole. Myocardial bridges are usually small and insignificant, and symptoms, such as tachycardia, usually appear during exertion. In some cases, profound MB symptoms include stable angina pain, unstable angina, atrioventricular block, and sudden death [53].

Myocardial bridging can also be single or multiple and is usually seen in the middle segment of the LAD rather than on the diagonal and marginal branches [54].

In the therapy of MB for symptomatic patients, β-blockers, which decrease the heart rate, remain the primary conservative treatment; calcium channel blockers, which have vasodilatory effects, are also common. Occasionally, stent implantation facilitates systolic coronary compression in symptomatic patients. Surgical intervention involves either supra-arterial myotomy or coronary artery bypass. Coronary artery bypass is indicated in patients with extensive (>25 mm) or deep (>5 mm) myocardial bridges [55].

A key limitation of this narrative review is the potential for selection bias, which arises both from our reliance on the existing literature and from the concept-driven selection of studies. Additionally, the representation of clinical cases and experiences drawn from our department may further influence the presentation and interpretation of findings. While MDCT offers numerous advantages in the evaluation of coronary artery anomalies (non-invasive, high spatial resolution, and three-dimensional/VR reconstruction capabilities), it is essential to acknowledge its limitations. One of the primary concerns associated with MDCT is radiation exposure, particularly in those requiring repeated imaging. Although recent advances in prospective ECG gating and dose-modulation techniques have significantly reduced radiation doses, exposure remains a relevant consideration. In addition, the use of iodinated contrast agents poses a risk of nephrotoxicity and allergic reactions, particularly in patients with pre-existing renal impairment. The slice thickness of the CT scan is also a critical factor in image quality and diagnostic accuracy, where thinner slices (like the 0.625 mm ones that we used) offer greater spatial resolution and are particularly useful for delineating the origin and course of anomalous coronary arteries. However, thinner slices also increase data volume and may contribute to longer processing times and greater computational demands. While invasive angiography remains the gold standard, it is limited in its ability to visualize the VR course of anomalous vessels, especially those with interarterial or intramural trajectories. In contrast, MDCT provides superior anatomical detail, making it particularly valuable in preoperative planning and risk stratification.

## 4. Conclusions

MDCT, volume rendering (VR), and maximum intensity projection techniques are valuable diagnostic tools for assessing coronary artery anomalies’ number, origin, and pathway. This is important, as the interpretations are essential for patients and physicians when considering further procedures and treatment plans. Furthermore, it allows the evaluation of the absence or presence of concomitant coronary artery diseases. This is particularly important for middle-aged populations.

## Figures and Tables

**Table 1:** Coronary vessels anatomy characteristics [6].

Morphological Component		Characteristic
**Artery root (ostium)**	**Number**	2 to 4 ostia
	**Origin**	Right and left aortic (Valsalva) sinuses
**Pathway**	**Orientation**	45° to 90° angulation to the aortic wall
	**Common stem**	Should exist only on the left side (from the left sinus)
	**Course**	Directly, following the anatomically designated path from the ostium to the destination territory of the myocardium
	**Localization**	Subepicardial

**Table 2:** Classification of atypical origin of coronary arteries.

Artery	Anomalous Origin	Course
**LMA**	Right sinus	In front of the PA
		Between the PA and the aortic bulb
**LAD**	Right sinus/RCA	In front of the PA
		Behind the aortic bulb
**LCX**	Right sinus/RCA	Behind the aortic bulb
**LCA**	The pulmonary artery (ALCAPA)	
**RCA**	Left sinus	Around the back of the PA, located on its front side
		Behind the aortic bulb
		Between the PA and the aortic bulb
	LMA (a single left trunk)	Around the TP from its back side to its front side
	The pulmonary artery (ARCAPA)	

LMA—left main coronary artery; LAD—left anterior descending artery; PA—pulmonary artery; LCX—left circumflex artery; LCA—left coronary artery; RCA—right coronary artery; ALCAPA—anomalous LCA from the pulmonary artery; ARCAPA—anomalous LCA from the pulmonary artery.

## Data Availability

No new data were created in this study.

## Abbreviations

The following abbreviations are used in this manuscript:

ALCAPA	Anomalous left coronary artery from the pulmonary artery
ARCAPA	Anomalous right coronary artery from the pulmonary artery
CA	Coronary artery
CABG	Coronary artery bypass grafting
LAD	Left anterior descending artery
LCX	Left circumflex artery
LMA	Left main artery
MB	Myocardial bridging
MDCT	Multidetector computed tomography
RCA	Right coronary artery
SCAD	Spontaneous coronary artery dissection
PA	Pulmonary artery
VR	Volume rendering

## References

[1] Szymczyk K., Polguj M., Szymczyk E., Majos A., Grzelak P., Stefańczyk L. (2014). Prevalence of Congenital Coronary Artery Anomalies and Variants in 726 Consecutive Patients Based on 64-Slice Coronary Computed Tomography Angiography. Folia Morphol..

[2] Gräni C., Benz D.C., Schmied C., Vontobel J., Possner M., Clerc O.F., Mikulicic F., Stehli J., Fuchs T.A., Pazhenkottil A.P. (2016). Prevalence and Characteristics of Coronary Artery Anomalies Detected by Coronary Computed Tomography Angiography in 5634 Consecutive Patients in a Single Centre in Switzerland. Swiss Med. Wkly..

[3] Graidis C., Dimitriadis D., Karasavvidis V., Dimitriadis G., Argyropoulou E., Economou F., George D., Antoniou A., Karakostas G. (2015). Prevalence and Characteristics of Coronary Artery Anomalies in an Adult Population Undergoing Multidetector-Row Computed Tomography for the Evaluation of Coronary Artery Disease. BMC Cardiovasc. Disord..

[4] Altin C., Kanyilmaz S., Koc S., Gursoy Y.C., Bal U., Aydinalp A., Yildirir A., Muderrisoglu H. (2015). Coronary Anatomy, Anatomic Variations and Anomalies: A Retrospective Coronary Angiography Study. Singapore Med. J..

[5] Angelini P. (2007). Coronary Artery Anomalies: An Entity in Search of an Identity. Circulation.

[6] Villa A.D., Sammut E., Nair A., Rajani R., Bonamini R., Chiribiri A. (2016). Coronary Artery Anomalies Overview: The Normal and the Abnormal. World J. Radiol..

[7] Beecham R., Prater S., Batlle J. (2025). Coronary Artery Anomalies. StatPearls.

[8] Gentile F., Castiglione V., De Caterina R. (2021). Coronary Artery Anomalies. Circulation.

[9] Czaja-Ziółkowska M., Głowacki J., Krysiński M., Gąsior M., Wasilewski J. (2023). Relationship between Left Main Trifurcation Angulation, Calcium Score, and the Onset of Plaque Formation. Kardiol. Pol..

[10] Earls J.P. (2006). Coronary Artery Anomalies. Tech. Vasc. Interv. Radiol..

[11] Tsang D.C., Link M.S. (2021). Sudden Cardiac Death in Athletes. Tex. Heart Inst. J..

[12] Bigler M.R., Stark A.W., Shiri I., Illi J., Siepe M., Caobelli F., Giannopoulos A.A., Buechel R.R., Haeberlin A., Obrist D. (2024). Noninvasive Anatomical Assessment for Ruling out Hemodynamically Relevant Coronary Artery Anomalies in Adults—A Comparison of Coronary-CT to Invasive Coronary Angiography: The NARCO Study Design. Contemp. Clin. Trials Commun..

[13] Nguyen V.T. (2019). MDCT in Diagnosis of Anomalies of Coronary Artery Origin and Course: A Coronary MDCT-Angiographic Study of 9572 Patients. Vasc. Dis. Ther..

[14] Helmy I., Nasr A., Ismail A., Ramadan A., Helmy K. (2012). Coronary Artery Anomalies: Role of Contrast Enhanced MDCT Coronary Angiography. Egypt. J. Radiol. Nucl. Med..

[15] Malik M.B., Zeltser R. (2025). Isolated Coronary Artery Anomalies. StatPearls.

[16] Yilmaz-Cankaya B., Kantarci M., Yalcin A., Durur-Karakaya A., Yuce I. (2009). Absence of the Left Main Coronary Artery: MDCT Coronary Angiographic Imaging. Eurasian J. Med..

[17] Duran C., Kantarci M., Durur Subasi I., Gulbaran M., Sevimli S., Bayram E., Eren S., Karaman A., Fil F., Okur A. (2006). Remarkable Anatomic Anomalies of Coronary Arteries and Their Clinical Importance: A Multidetector Computed Tomography Angiographic Study. J. Comput. Assist. Tomogr..

[18] Ashraf S., Salman S.H., Ali N., Kulshreshtha S., Saad M. (2020). A Rare Presentation of Angina and Arrhythmia in Absent Left Main Coronary Artery. Cureus.

[19] Mohsen G.A., Mohsin K.G., Forsberg M., Miller E., Taniuchi M., Klein A.J.P. (2013). Anomalous Left Circumflex Artery from the Right Coronary Cusp: A Benign Variant?. J. Invasive Cardiol..

[20] Rissam H.K., Garg L., Mittal U.K., Singh S. (2015). Uncommon Variants of Left Circumflex Coronary Artery (LCX): Evaluation with 256-Slice Dual Source CT Coronary Angiography. BMJ Case Rep..

[21] White N.K., Edwards J.E. (1948). Anomalies of the Coronary Arteries; Report of Four Cases. Arch. Pathol..

[22] Yildirim E., Yuksel U.C., Bugan B., Celik M., Gokoglan Y., Bozlar U. (2016). Malignant Right Coronary Artery Originating from Left Coronary Sinus. Int. J. Cardiovasc. Acad..

[23] Narayanan S.R., Al Shamkhani W., Rajappan A.K. (2016). Anomalous Origin of RCA from Left Coronary Sinus Presenting as PSVT and Recurrent Acute Coronary Syndromes. Indian Heart J..

[24] Angelini P., Trujillo A., Sawaya F., Lee V.-V. (2008). “Acute Takeoff” of the Circumflex Artery: A Newly Recognized Coronary Anatomic Variant with Potential Clinical Consequences. Tex. Heart Inst. J..

[25] Cong M., Zhao H., Dai S., Chen C., Xu X., Qiu J., Qin S. (2021). Transient Numerical Simulation of the Right Coronary Artery Originating from the Left Sinus and the Effect of Its Acute Take-off Angle on Hemodynamics. Quant. Imaging Med. Surg..

[26] Loukas M., Andall R.G., Khan A.Z., Patel K., Muresian H., Spicer D.E., Tubbs R.S. (2016). The Clinical Anatomy of High Take-off Coronary Arteries. Clin. Anat..

[27] Terlemez S., Kula S., Oğuz D. (2021). High Take-off Right Coronary Artery in a Patient with Tetralogy of Fallot. Cardiol. Young.

[28] Deng X., Huang P., Chen W., Yang X., Liu Q., Xiao Y., He C. (2017). An Incidental Encounter of a Rare High Take-off Right Coronary Artery: A Case Report. Medicine.

[29] Hyrtl (1841). Varieties in the Distribution of Vessels Interesting to the Surgeon. Prov. Med. Surg. J..

[30] Smith J.C. (1950). Review of Single Coronary Artery with Report of 2 Cases. Circulation.

[31] Anandi S., Ghosh D., Roy S., Ahuja M.S., Bandyopadhyay D. (2024). Anatomical Diversification of Left Coronary Artery and Its Branches in Western Maharashtra Population. Natl. J. Clin. Anat..

[32] Ajayi N.O., Lazarus L., Vanker E.A., Satyapal K.S. (2013). The Prevalence and Clinical Importance of an “Additional” Terminal Branch of the Left Coronary Artery. Folia Morphol..

[33] Khachatryan A., Chow R.T., Srivastava M.C., Cinar T., Alejandro J., Sargsyan M., Shaik M.R., Tamazyan V., Haque R.U., Harutyunyan H. (2024). The Ramus Intermedius: A Bridge to Survival in the Setting of Triple-Vessel Total Occlusion. Cureus.

[34] Rosani N.S., Zamin R.M., Aman R.R.A.R., Zuhdi A.S.M., Danaee M., Zulkafli I.S. (2025). The Influence of the Presence of the Ramus Intermedius on Atherosclerosis Plaque Deposition in the Left Bifurcation Region in Low-Risk Individuals. Rev. Cardiovasc. Med..

[35] Michałowska I.M., Hryniewiecki T., Kwiatek P., Stokłosa P., Swoboda-Rydz U., Szymański P. (2016). Coronary Artery Variants and Anomalies in Patients With Bicuspid Aortic Valve. J. Thorac. Imaging.

[36] Mishra D., Raj G., Singh B., Mishra D., Nayak R.K. (2021). Prevalence and Distribution of Coronary Dominance and Ramus Intermedius in North Indian Population on CT Coronary Angiography—A Cross-Sectional Study. Int. J. Anat. Radiol. Surg..

[37] Kovacevic M., Burzotta F., Elharty S., Besis G., Aurigemma C., Romagnoli E., Trani C. (2021). Left Main Trifurcation and Its Percutaneous Treatment: What Is Known So Far?. Circ. Cardiovasc. Interv..

[38] Kandzari D.E., Gershlick A.H., Serruys P.W., Leon M.B., Morice M.-C., Simonton C.A., Lembo N.J., Mansour S., Sabaté M., Sabik J.F. (2020). Procedural Characteristics and Clinical Outcomes in Patients Undergoing Percutaneous Coronary Intervention for Left Main Trifurcation Disease: The EXCEL Trial. EuroIntervention.

[39] Gong X., Huang Z., Sun Z., Wang Q., Qian J., Ge L., Ge J. (2021). Role of IVUS in the Rectification of Angiographically Judged Ramus Intermedius and Its Clinical Significance. BMC Cardiovasc. Disord..

[40] Misuraca L., Balbarini A. (2010). Double Right Coronary Artery or Split Right Coronary Artery: The Same Anomaly?. J. Cardiovasc. Med. Hagerstown Md.

[41] Sato Y., Kunimasa T., Matsumoto N., Saito S. (2008). Detection of Double Right Coronary Artery by Multi-Detector Row Computed Tomography: Is Angiography Still Gold Standard?. Int. J. Cardiol..

[42] Naik S., Bansal R. (2021). Dual versus Split Right Coronary Artery: What Is There in the Name?. Int. J. Cardiovasc. Acad..

[43] Chien T.-M., Chen C.-W., Chen H.-M., Lee C.-S., Lin C.-C., Chen Y.-F. (2014). Double Right Coronary Artery and Its Clinical Implications. Cardiol. Young.

[44] Sim D.S., Jeong M.H., Choi S., Yoon N.S., Yoon H.J., Moon J.Y., Hong Y.J., Kim K.H., Park H.W., Kim J.H. (2009). Myocardial Infarction in a Young Man Due to a Hypoplastic Coronary Artery. Korean Circ. J..

[45] Amabile N. (2005). Hypoplastic Coronary Artery Disease: Report of One Case. Heart.

[46] Kurt I.H. (2009). Coronary Artery Disease Mimicking Tako-Tsubo Cardiomyopathy: A Case Report. Cases J..

[47] Riede F.-N., Bulla S., Grundmann S., Werner M., Riede U.-N., Otto C. (2013). Isolated Hypoplastic Circumflex Coronary Artery: A Rare Cause of Haemorrhagic Myocardial Infarction in a Young Athlete. Diagn. Pathol..

[48] Wenger N.K., Peace R.J. (1961). Rudimentary Left Coronary Artery∗. Am. J. Cardiol..

[49] Roberts W.C., Glick B.N. (1992). Congenital Hypoplasia of Both Right and Left Circumflex Coronary Arteries. Am. J. Cardiol..

[50] Hassan A.K.M., Abd-El Rahman H., Hassan S.G., Ahmed T.A.N., Youssef A.A.A. (2018). Validity of Tortuosity Severity Index in Chest Pain Patients with Abnormal Exercise Test and Normal Coronary Angiography. Egypt. Heart J. EHJ Off. Bull. Egypt. Soc. Cardiol..

[51] Eleid M.F., Guddeti R.R., Tweet M.S., Lerman A., Singh M., Best P.J., Vrtiska T.J., Prasad M., Rihal C.S., Hayes S.N. (2014). Coronary Artery Tortuosity in Spontaneous Coronary Artery Dissection: Angiographic Characteristics and Clinical Implications. Circ. Cardiovasc. Interv..

[52] Santucci A., Jacoangeli F., Cavallini S., d’Ammando M., de Angelis F., Cavallini C. (2022). The Myocardial Bridge: Incidence, Diagnosis, and Prognosis of a Pathology of Uncertain Clinical Significance. Eur. Heart J. Suppl. J. Eur. Soc. Cardiol..

[53] Sylvia M.T., Soundharia R., Bhat R.V., Marak F. (2023). Myocardial Bridging in Cases of Sudden Death and Its Association with Clinicopathologic Characteristics. Heart Views Off. J. Gulf Heart Assoc..

[54] Yuan S.-M. (2016). Myocardial Bridging. Braz. J. Cardiovasc. Surg..

[55] Sternheim D., Power D.A., Samtani R., Kini A., Fuster V., Sharma S. (2021). Myocardial Bridging: Diagnosis, Functional Assessment, and Management. J. Am. Coll. Cardiol..

